# Survival among subgroups of patients with stage II nasopharyngeal carcinoma

**DOI:** 10.1038/s41598-022-11145-4

**Published:** 2022-04-29

**Authors:** Shi-Ting Huang, Dan-Ke Su

**Affiliations:** 1 Department of Radiation Oncology, Guangxi Medical University Cancer Hospital, Nanning, 530021 Guangxi People’s Republic of China; 2 Department of Radiology, Guangxi Medical University Cancer Hospital, Qingxiu District, No. 71 Hedi Road, Nanning, 530021 Guangxi People’s Republic of China

**Keywords:** Cancer, Oncology

## Abstract

To assess survival between subgroups (T1N1, T2N0, and T2N1) of patients with stage II nasopharyngeal carcinoma (NPC). This retrospective cohort study evaluated pathologically confirmed stage II NPC patients from The Surveillance, Epidemiology, and End Results (SEER) database from 2004 to 2016. The included patients were divided into three subgroups: T1N1, T2N0, and T2N1. Overall survival (OS) and cancer-specific survival (CSS) were assessed using the Kaplan–Meier method among the three subgroups. This study investigated 836 patients: 383 (45.8%) patients were in the T1N1 subgroup, 175 (20.9%) patients were in the T2N0 subgroup, and 278 (33.3%) patients were in the T2N1 subgroup. The 5-year OS (75.7%, 68.6%, and 75.7%) and CSS (85.3%, 83.4%, and 84.5%) were similar among the T1N1, T2N0, and T2N1 subgroups. Univariate and multivariate regression analyses revealed that the subgroup (T1N1, T2N0, and T2N1) of stage II NPC was not an independent prognostic factor for OS or CSS. Survival was comparable among subgroups (T1N1, T2N0, and T2N1) of stage II NPC patients. However, patients with T1N1, T2N0, and T2N1 stage disease who receive different treatments might have different prognoses.

## Introduction

Nasopharyngeal carcinoma (NPC) is a common head and neck cancer in southern China^1^. The incidence of NPC was reported to be 3.0 per 100,000 person-years^2,3^. Due to its unique biological characteristics and covert location, most cases are diagnosed with loco-regionally advanced NPC. However, the incidence of stage II NPC has greatly increased with improvements in diagnosis.

Stage II NPC is divided into three subgroups (T1N1, T2N0, and T2N1). It has been reported that patients with stage T1N1 and T2N1 disease might benefit from chemotherapy, while radiotherapy could provide an excellent outcome for stage T2N0 disease^4–6^. These studies indicated that the prognosis among these three subgroups might be different. Thus, the Chinese Anti-Cancer Association recommended RT alone for the T2N0 subgroup and RT with or without chemotherapy for the T1N1 and T2N1 subgroups^7^. In contrast, several studies have suggested that survival was similar among the T1N1, T2N0, and T2N1 subgroups^8–11^. Thus, chemoradiotherapy with or without adjuvant chemotherapy is recommended for stage II NPC by the National Comprehensive Cancer Network^12^.

To date, the survival outcomes among the T1N1, T2N0, and T2N1 subgroups of stage II NPC have not been well defined. Treatment modalities of stage II NPC vary among different centers. This retrospective cohort study was conducted to assess survival among the T1N1, T2N0, and T2N1 subgroups of patients with stage II NPC based on the Surveillance, Epidemiology, and End Results (SEER) database. The results might provide novel information on the prognosis of stage II NPC, which might be used to improve treatment modalities.

## Materials and methods

### Patient selection

NPC patients were extracted from the SEER database from 2004 to 2016. The inclusion criteria were as follows: (1) pathologically confirmed NPC, (2) stage II patients, (3) patients who received radiotherapy, and (4) patients with definite information on race and pathology classification. Patient characteristics, including age, sex, race, World Health Organization (WHO) classification, and chemotherapy, were extracted. The included patients were divided into T1N1, T2N0, and T2N1 subgroups.

This study was based on the SEER database. Ethical approval was waived by Guangxi Medical University Cancer Hospital. All experiments adhered to relevant ethical guidelines for handling human data.

### Endpoints

Overall survival (OS) was the primary endpoint. OS was defined as the time from diagnosis to death as a result of any cause. Cancer-specific survival (CSS) was the secondary endpoint. CSS was defined as the time from diagnosis to death attributed to NPC.

### Statistical analysis

Continuous characteristics of age were compared using Student’s t test. Categorical variables of sex, race, WHO classification, and chemotherapy were analyzed by using the χ^2^ test or Fisher's exact test.

The 5-year OS and CSS rates of the T1N1, T2N0, and T2N1 subgroups were calculated using Kaplan–Meier analysis. Differences between survival curves were compared using the log-rank test. Univariate regression analysis was performed to identify prognostic factors. Multivariable proportional hazards models adjusted for age, sex, race, WHO classification, and chemotherapy were performed to assess independent prognostic factors. The results are reported as hazard ratios (HRs) with 95% confidence intervals (CIs).

All statistical analyses were performed using SPSS Statistics Version 26.0 software (IBM Co., Armonk, NY, USA) and R software version 4.0.3 (http://www.R-project.org). P values were two-tailed. Values of *P* < 0.05 were considered statistically significant.

## Results

### Patient characteristics

**Figure 1:**
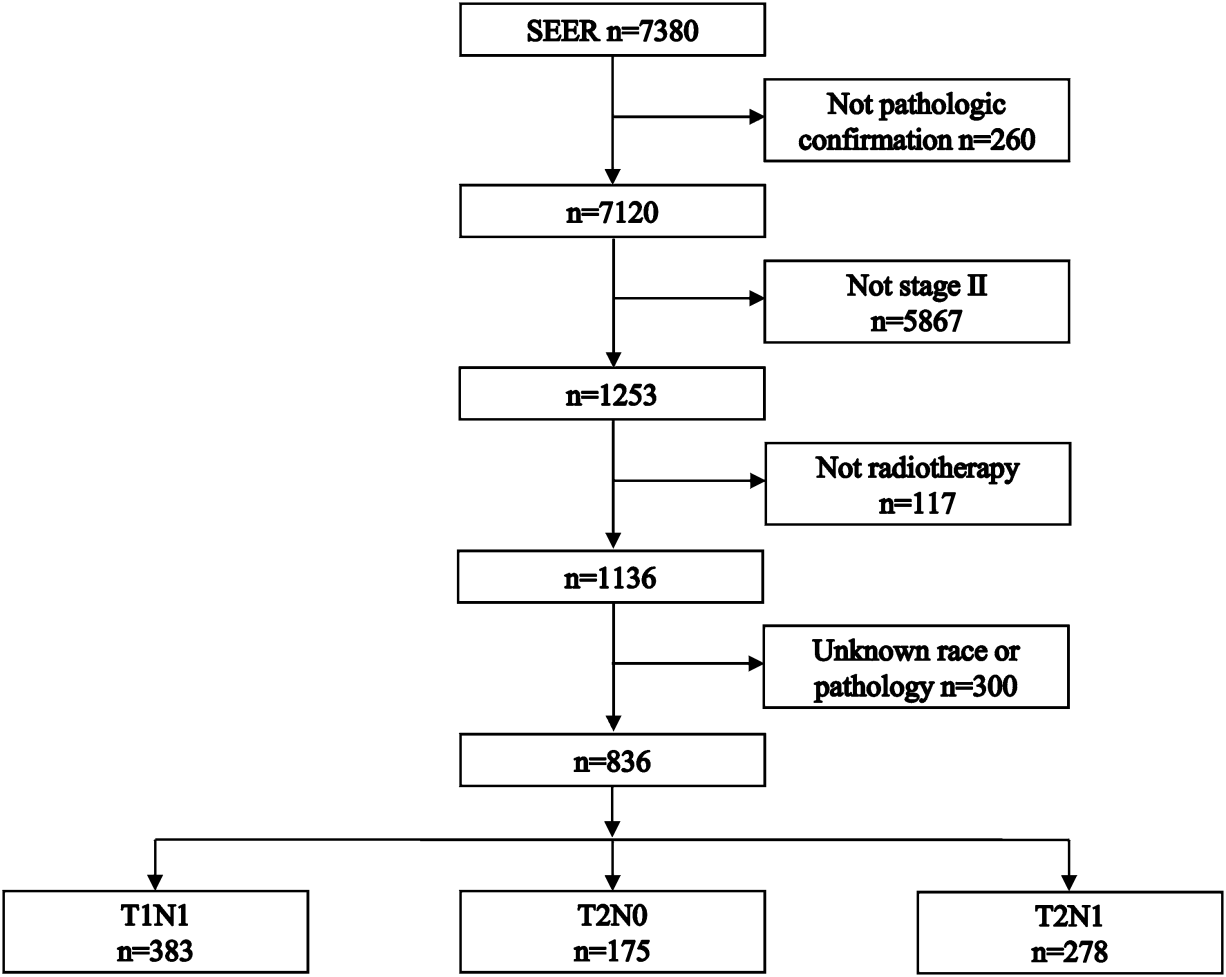
Flowchart of patient selection.

The patient selection flowchart is shown in Fig. 1. This retrospective cohort study included 836 stage II NPC patients. The T1N1 subgroup included 383 (45.8%) patients. The T2N0 subgroup included 175 (20.9%) patients. The T2N1 subgroup included 278 (33.3%) patients. The baseline characteristics are listed in Table 1.

**Table 1:** Patient characteristics.

	T1N1 (n = 383)	T2N0 (n = 175)	T2N1 (n = 278)	*P*
**Age (year)**				
Median	56	59	55	< 0.01
IQR	46–64	51–68	46–64	
**Sex**				0.27
Male	254 (66.3%)	125 (71.4%)	199 (71.6%)	
Female	129 (33.7%)	50 (28.6%)	79 (28.4%)	
**Race**				< 0.01
Asian	152 (39.7%)	52 (29.7%)	105 (37.8%)	
Black	29 (7.6%)	29 (16.6%)	22 (7.9%)	
White	202 (52.7%)	94 (53.7%)	151 (54.3%)	
**Pathology**				0.14
WHO I	172 (44.9%)	89 (50.9%)	115 (41.4%)	
WHO II/III	211 (55.1%)	86 (49.1%)	163 (58.6%)	
**Chemotherapy**				< 0.01
No	33 (8.6%)	43 (24.6%)	14 (5.0%)	
Yes	350 (91.4%)	132 (75.4%)	264 (95.0%)	

*IQR* interquartile range, *WHO* World Health Organization.

### Survival analysis of the T1N1, T2N0, and T2N1 subgroups

**Figure 2:**
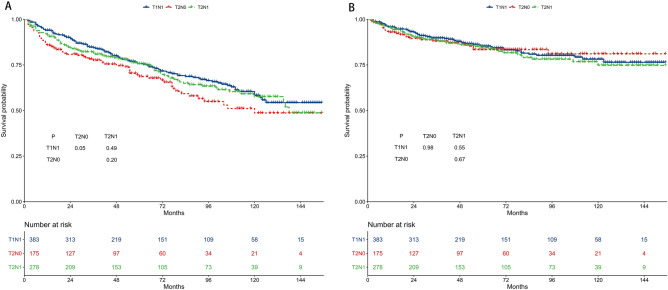
Survival outcomes of the T1N1, T2N0, and T2N1 subgroups. (**A**) Overall survival. (**B**) Cancer-specific survival.

The 5-year OS rates were 75.7%, 68.6%, and 75.7% for the T1N1, T2N0, and T2N1 subgroups, respectively (Fig. 2A). The 5-year OS of the three groups was not different in pairwise comparisons. The 5-year CSS rates were 85.3%, 83.4%, and 84.5% for the T1N1, T2N0, and T2N1 subgroups, respectively (Fig. 2B). The 5-year CSS of the three groups was not different.

Univariate regression analysis revealed that age, race, WHO classification, and chemotherapy were prognostic factors for OS. On the other hand, age and WHO classification were prognostic factors for CSS. However, the subgroup (T1N1, T2N0, and T2N1) was not a prognostic factor for OS and CSS (Table 2).

**Table 2:** Univariate regression analysis of overall survival and cancer-specific survival.

	Overall survival			Cancer-specific survival		
	HR	95% CI	*P*	HR	95% CI	*P*
Age	1.05	1.04–1.06	< 0.01	1.03	1.02–1.07	< 0.01
**Sex**						
Male	Reference			Reference		
Female	0.83	0.64–1.09	0.18	0.75	0.51–1.11	0.14
**Race**						
Asian	Reference			Reference		
Black	1.94	1.24–3.02	< 0.01	1.53	0.81–2.88	0.19
White	1.99	1.49–2.65	< 0.01	1.62	1.10–2.40	0.15
**Pathology**						
WHO I	Reference			Reference		
WHO II/III	0.47	0.36–0.60	< 0.01	0.60	0.43–0.85	< 0.01
**Chemotherapy**						
No	Reference			Reference		
Yes	0.60	0.42–0.84	< 0.01	0.87	0.50–1.52	0.63
**Group**						
T1N1	Reference			Reference		
T2N0	1.36	1.00–1.86	0.05	1.01	0.63–1.61	0.98
T2N1	1.11	0.83–1.46	0.49	1.12	0.76–1.65	0.55

*WHO* World Health Organization, *HR* hazard ratio, *CI* confidence interval.

**Figure 3:**
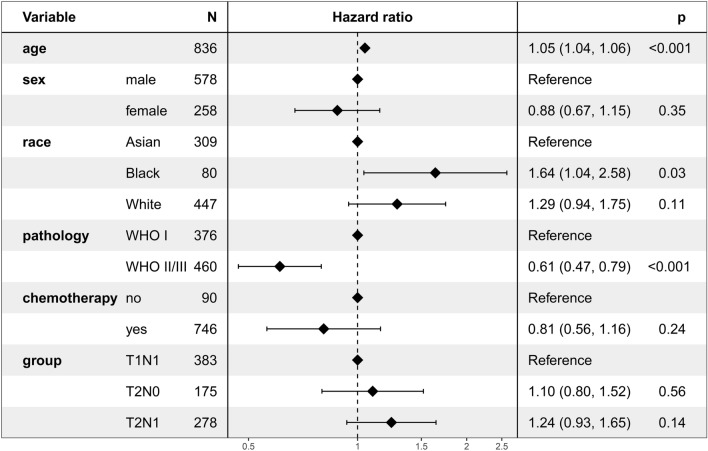
Multivariate regression analysis of the prognostic factors for overall survival.

Multivariate Cox regression analysis revealed that the subgroup (T1N1, T2N0, and T2N1) was not an independent prognostic factor for OS (Fig. 3). Setting the T1N1 subgroup as a reference, the HR was 1.10 (95% CI 0.80–1.52; *P* = 0.56) and 1.24 (95% CI 0.93–1.65; *P* = 0.14) for the T2N0 and T2N1 subgroups, respectively.

**Figure 4:**
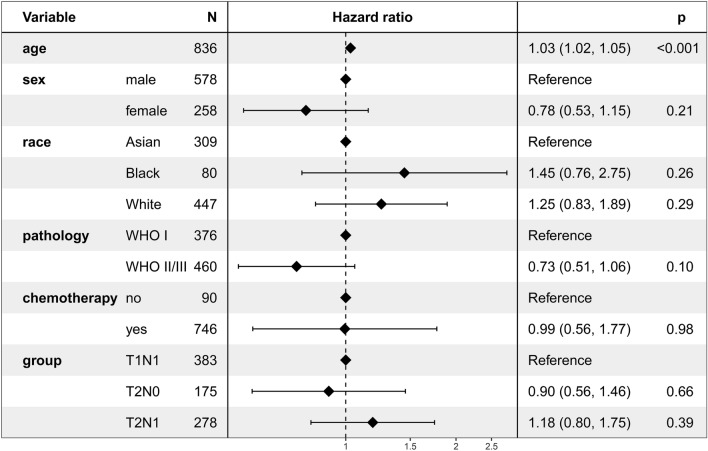
Multivariate regression analysis of the prognostic factors for cancer-specific survival.

Multivariate Cox regression analysis revealed that the subgroup (T1N1, T2N0, and T2N1) was not an independent prognostic factor for CSS (Fig. 4). Setting the T1N1 subgroup as a reference, the HR was 0.90 (95% CI 0.56–1.46; *P* = 0.66) and 1.18 (95% CI 0.80–1.75; *P* = 0.39) for the T2N0 and T2N1 subgroups***, respectively.***

### Survival of the T1N1, T2N0, and T2N1 subgroups in the chemoradiotherapy group

**Figure 5:**
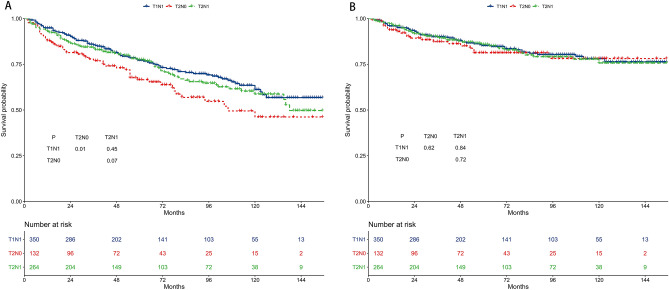
Survival outcomes of the T1N1, T2N0, and T2N1 subgroups in the chemoradiotherapy group. (**A**) Overall survival. (**B**) Cancer-specific survival.

The 5-year OS rates were 77.0%, 69.7%, and 74.6% for the T1N1, T2N0, and T2N1 subgroups, respectively (Fig. 5A). The 5-year OS was worse in the T2N0 subgroup than in the T1N1 subgroup (*P* = 0.01). The 5-year CSS rates were 84.5%, 83.9%, and 83.6% for the T1N1, T2N0, and T2N1 subgroups, respectively (Fig. 5B). The 5-year CSS of the three subgroups was not different.

### Survival of the T1N1, T2N0, and T2N1 subgroups in the radiotherapy group

**Figure 6:**
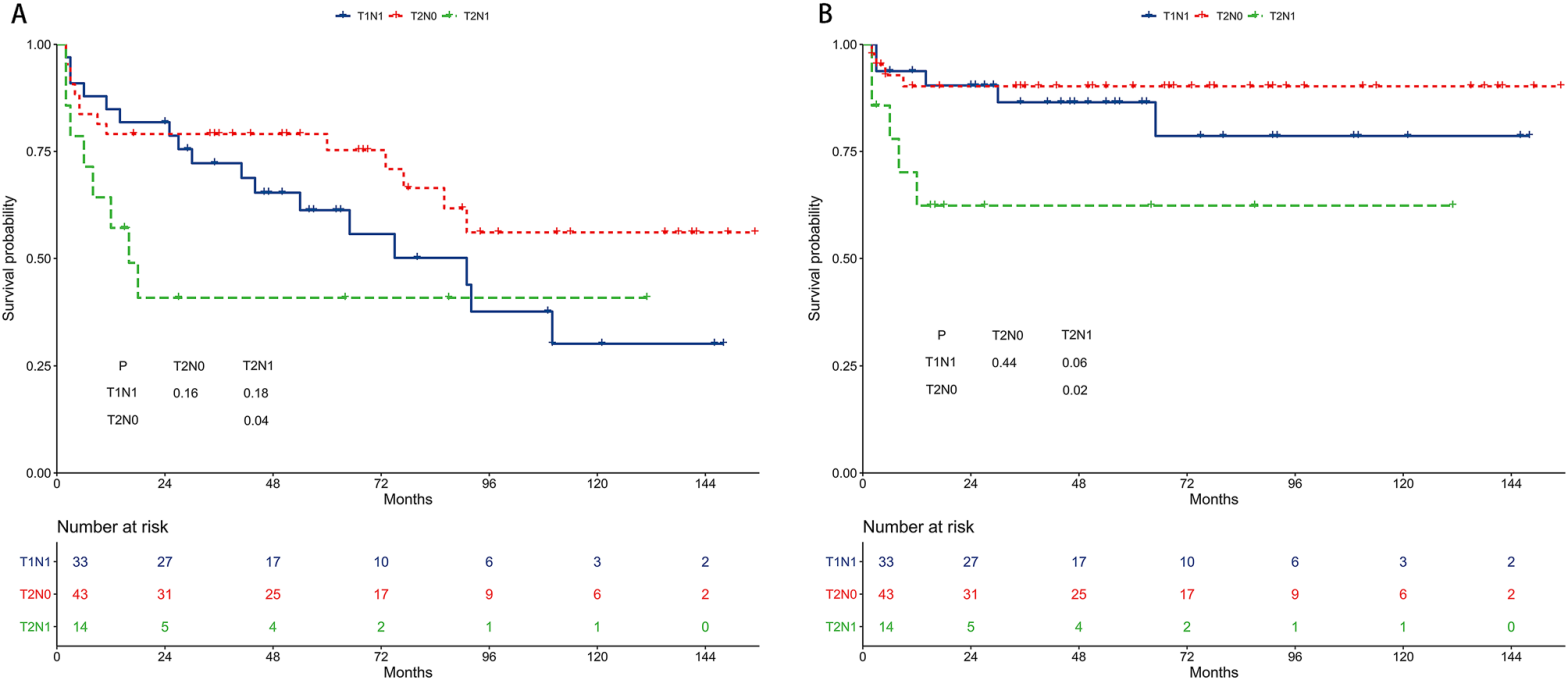
Survival outcomes of the T1N1, T2N0, and T2N1 subgroups in the radiotherapy group. (**A**) Overall survival. (**B**) Cancer-specific survival.

The 5-year OS rates were 67.6%, 69.9%, and 63.0% for the T1N1, T2N0, and T2N1 subgroups, respectively (Fig. 6A). The 5-year OS was worse in the T2N1 subgroup than in the T2N0 subgroup (*P* = 0.04). The 5-year CSS rates were 88.9%, 84.7%, and 76.6% for the T1N1, T2N0, and T2N1 subgroups, respectively (Fig. 6B). The 5-year CSS was worse in the T2N1 subgroup than in the T2N0 subgroup (*P* = 0.02).

### Survival of the T1N1, T2N0, and T2N1 subgroups in the WHO I group

**Figure 7:**
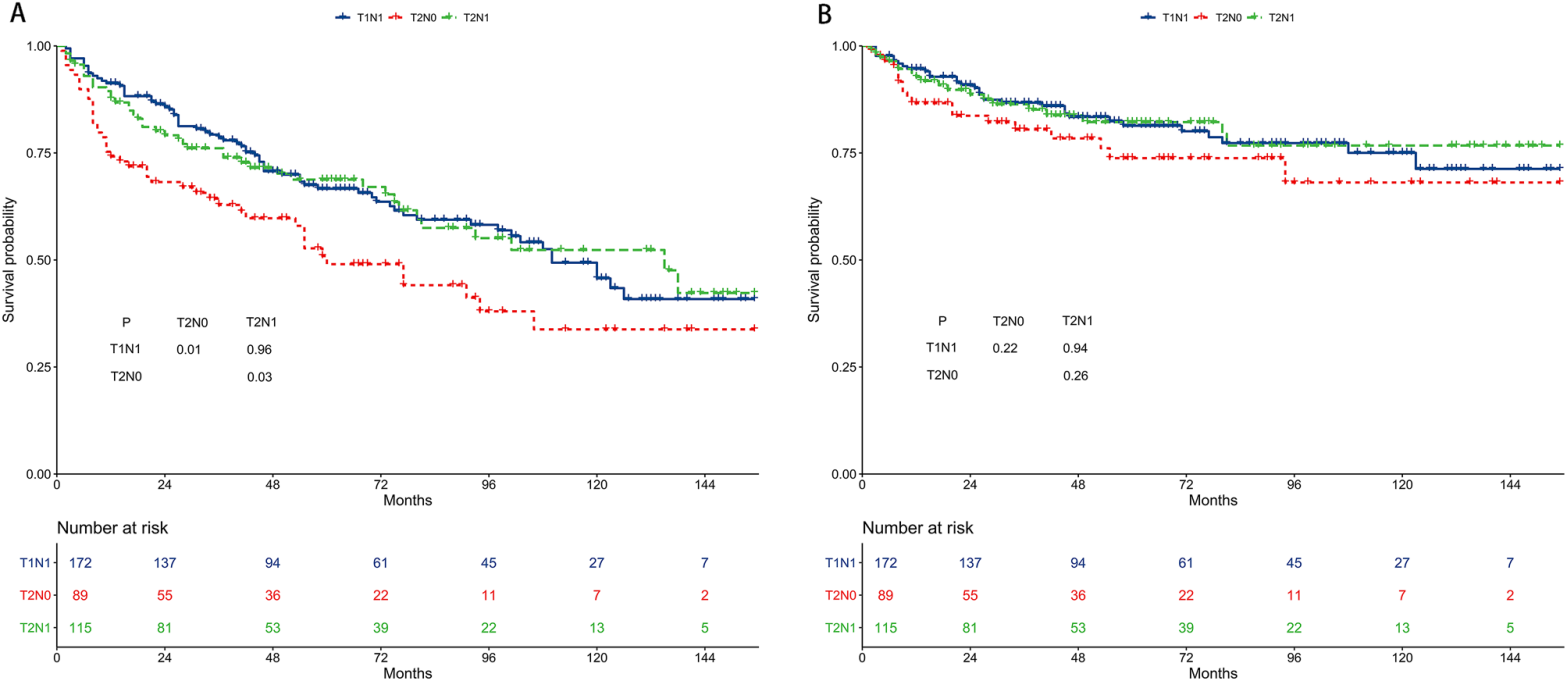
Survival outcomes of the T1N1, T2N0, and T2N1 subgroups in the WHO I group. (**A**) Overall survival. (**B**) Cancer-specific survival.

The 5-year OS rates were 67.1%, 49.0%, and 68.7% for the T1N1, T2N0, and T2N1 subgroups, respectively (Fig. 7A). The T1N1 and T2N1 subgroups had better 5-year OS than the T2N0 subgroup. The 5-year CSS rates were 81.6%, 73.3%, and 82.6% for the T1N1, T2N0, and T2N1 subgroups, respectively (Fig. 7B). The 5-year CSS of the three subgroups was not different.

### Survival of the T1N1, T2N0, and T2N1 subgroups in the WHO II/III group

**Figure 8:**
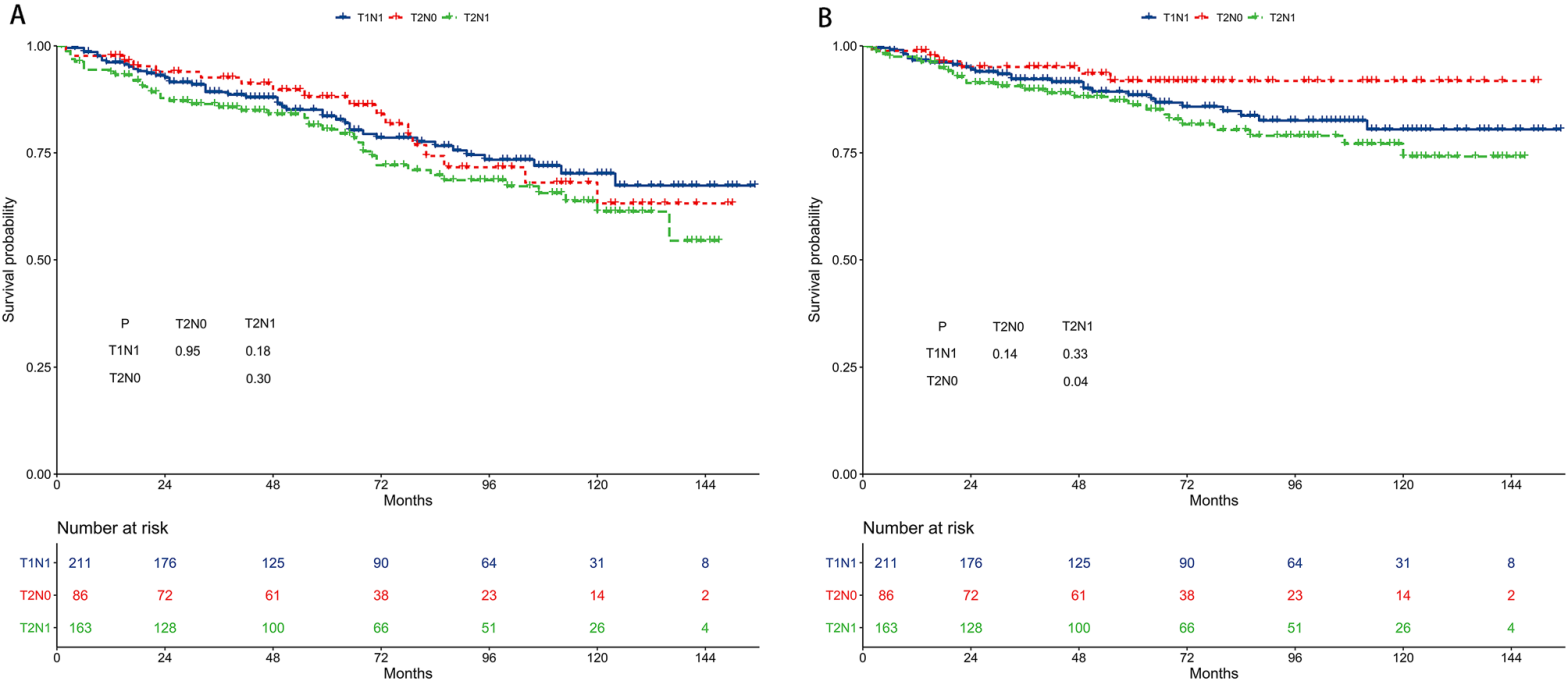
Survival outcomes of the T1N1, T2N0, and T2N1 subgroups in the WHO II/III group. (**A**) Overall survival. (**B**) Cancer-specific survival.

The 5-year OS rates were 83.6%, 88.3%, and 80.4% for the T1N1, T2N0, and T2N1 subgroups, respectively (Fig. 8A). The 5-year OS of the three subgroups was not different. The 5-year CSS rates were 88.6%, 92.0%, and 86.6% for the T1N1, T2N0, and T2N1 subgroups, respectively (Fig. 8B). The 5-year CSS was worse in the T2N1 subgroup than in the T2N0 subgroup (*P* = 0.04).

## Discussion

It has been reported that the survival outcomes differ among patients with T1N1, T2N0, and T2N1 stage II NPC. Several studies have suggested that the T2N1 subgroup might be a unique subgroup with worse survival outcomes^4,13–15^. The worse survival outcomes in the T2N1 subgroup might be due to the following: First, parapharyngeal extension increased the risk of distant metastasis in stage II NPC patients^16–18^. The 5-year distant metastasis-free survival with parapharyngeal extension was 12.6% lower than that in patients without parapharyngeal extension (73.6% vs. 86.2%)^18^. Moreover, the 5-year distant metastasis-free survival of stage T1 was significantly higher than that of stage T2 for patients with stage N1^19^. Second, several studies suggested that stage N1 was an independent prognostic risk factor^14,20–22^.

In contrast, several studies have suggested that survival is similar among subgroups of stage II NPC^8–11^. Similarly, our study also revealed that the survival curves were comparable among the T1N1, T2N0, and T2N1 subgroups for OS and CSS. However, the T2N1 subgroup was more likely to receive chemotherapy (95.0%) in our study. This raised the question of whether chemotherapy increased the survival rate of the T2N1 subgroup. Survival analysis showed that chemotherapy improved 5-year OS (77.5% vs. 40.8%, *P* < 0.01) and CSS (85.8% vs. 62.3%, *P* < 0.01) in the T2N1 subgroup. Although there was an improvement in survival resulting from chemotherapy in the T2N1 subgroup, there were no survival differences among the T1N1, T2N0, and T2N1 subgroups.

However, some studies have reported that chemotherapy did not improve the survival of the T2N1 subgroup. A previous study indicated that radiotherapy alone provided comparable 5-year OS (77.19% vs. 68.28%; *P* = 0.06) in the T2N1 subgroup compared with chemoradiotherapy^23^. Moreover, other studies also found that chemoradiotherapy did not improve OS compared to radiotherapy alone in the T2N1 subgroup^9,11,15^. The differences among these studies might be due to the small sample size of the radiotherapy-alone subgroup. Similarly, the sample size of patients who received radiotherapy alone was extremely small (10.8%) in our study, so it might not be sufficient for the statistical analysis. Although the results indicated that the 5-year OS and CSS were worse in the T2N1 subgroup than in the T2N0 subgroup, the result should be verified in another cohort with a large sample size.

The results of our study should be interpreted with caution. Tumors that invaded the medial pterygoid muscle and the lateral pterygoid muscle without other T3/T4 involvement were downstaged from stage T4 to T2 according to the 8th edition AJCC staging system^24,25^. It is possible that survival among subgroups of stage II NPC patients might be different according to the 8th edition AJCC staging system. Due to the limitations of the SEER database, data on medial pterygoid muscle/lateral pterygoid muscle involvement could not be extracted. Our retrospective study could not assess survival among subgroups of stage II NPC according to the 8th edition AJCC staging system. Further studies are needed to verify the results of our study based on the 8th edition AJCC staging system.

The limitations of this study should be noted. First, data on radiotherapy techniques could not be extracted given the limitations of the SEER database. It was reported that intensity-modulated radiation therapy was superior to two-dimensional conventional radiotherapy^26,27^. However, other studies have found no difference in OS between intensity-modulated radiation therapy and two-dimensional conventional radiotherapy^28–30^. Thus, the different radiotherapy techniques used in this study might not have impacted the results. Second, Epstein–Barr virus (EBV) DNA is reported to be a prognostic factor for NPC^31–34^. Chen et al.^35^ reported that EBV DNA could improve the prognostic stratification of stage II NPC. However, EBV DNA data were not available due to the limitations of the SEER database in our study. Thus, our study could not assess the prognostic value of EBV DNA in stage II NPC. Third, we could not extract data on chemotherapy regimens and drugs. Thus, our study did not assess the impact of chemotherapy regimens on survival due to the lack of chemotherapy information.

In conclusion, the results of this retrospective cohort study suggested that survival among subgroups (T1N1, T2N0, and T2N1) of patients with stage II NPC was comparable based on the SEER database. However, subgroup analysis indicated that patients with T1N1, T2N0, and T2N1 stages receiving different treatments might have different prognoses. Further studies with large sample sizes are needed to identify prognostic risk factors for stage II NPC.
